# lncRNAfunc: a knowledgebase of lncRNA function in human cancer

**DOI:** 10.1093/nar/gkab1035

**Published:** 2021-11-17

**Authors:** Mengyuan Yang, Huifen Lu, Jiajia Liu, Sijia Wu, Pora Kim, Xiaobo Zhou

**Affiliations:** West China Biomedical Big Data Center, West China Hospital, Sichuan University, Chengdu 610041, China; Med-X Center for Informatics, Sichuan University, Chengdu 610041, China; Center for Computational Systems Medicine, School of Biomedical Informatics, The University of Texas Health Science Center at Houston, Houston, TX 77030, USA; West China Biomedical Big Data Center, West China Hospital, Sichuan University, Chengdu 610041, China; Med-X Center for Informatics, Sichuan University, Chengdu 610041, China; Center for Computational Systems Medicine, School of Biomedical Informatics, The University of Texas Health Science Center at Houston, Houston, TX 77030, USA; College of Electronic and Information Engineering, Tongji University, Shanghai, Shanghai 201804, China; School of Life Sciences and Technology, Xidian University, Xi’an 710126, China; Center for Computational Systems Medicine, School of Biomedical Informatics, The University of Texas Health Science Center at Houston, Houston, TX 77030, USA; Center for Computational Systems Medicine, School of Biomedical Informatics, The University of Texas Health Science Center at Houston, Houston, TX 77030, USA; McGovern Medical School, The University of Texas Health Science Center at Houston, Houston, TX 77030, USA; School of Dentistry, The University of Texas Health Science Center at Houston, Houston, TX 77030, USA

## Abstract

The long non-coding RNAs associating with other molecules can coordinate several physiological processes and their dysfunction can impact diverse human diseases. To date, systematic and intensive annotations on diverse interaction regulations of lncRNAs in human cancer were not available. Here, we built lncRNAfunc, a knowledgebase of lncRNA function in human cancer at https://ccsm.uth.edu/lncRNAfunc, aiming to provide a resource and reference for providing therapeutically targetable lncRNAs and intensive interaction regulations. To do this, we collected 15 900 lncRNAs across 33 cancer types from TCGA. For individual lncRNAs, we performed multiple interaction analyses of different biomolecules including DNA, RNA, and protein levels. Our intensive studies of lncRNAs provide diverse potential mechanisms of lncRNAs that regulate gene expression through binding enhancers and 3′-UTRs of genes, competing for miRNA binding sites with mRNAs, recruiting the transcription factors to gene promoters. Furthermore, we investigated lncRNAs that potentially affect the alternative splicing events through interacting with RNA binding Proteins. We also performed multiple functional annotations including cancer stage-associated lncRNAs, RNA A-to-I editing event-associated lncRNAs, and lncRNA expression quantitative trait loci. lncRNAfunc is a unique resource for cancer research communities to help better understand potential lncRNA regulations and therapeutic lncRNA targets.

## INTRODUCTION

lncRNAs are conserved RNAs with a length of >200 nucleotides that can regulate the expression of target genes with diverse cellular mechanisms including signal, decoy, guide, and scaffold (1–3). Recently, there have been found large numbers of lncRNAs that play roles in regulating cancer development and progression by interacting with multiple biomolecules. For example, one of the famous lncRNAs, metastasis-associated lung adenocarcinoma transcript 1 (*MALAT1*) is a drug targetable lncRNA (4). *MALAT1* interaction with the enhancer of zeste homolog 2 (EZH2) suppresses E-cadherin expression, thus promoting osteosarcoma metastasis. Furthermore, *MALAT1* facilitated the EZH2-mediated prostate cancer cell invasion by interacting with the promoter of *EZH2* (5,6). These examples show that studying the lncRNA interactions can provide key mechanisms of cellular processes related to tumorigenesis.

From the emerging importance of lncRNAs in cancer, studying upstream regulators and downstream target genes related to lncRNAs is essential to fully understand the cancer progression and identify the therapy targets. There is much evidence showing that abnormal lncRNA expression accompanies DNA damage, immune escape as well as cellular metabolic disorders in cancer cells (7). The diverse types of interaction of lncRNAs with different biomolecules may be related to the complicated tumorigenesis (8). To our knowledge, there are four databases (LncRNA2Target (9), LncRRIsearch (10), LncTarD (11), and NPInter (12)) providing the interaction annotation of lncRNAs. However, these studies lack detailed and intensive downstream analyses for individual interaction relationships, which is not enough to fully understand the lncRNAs’ mechanisms of action in cancer. LncReg (13) and LncExpDB (14) also only provide limited analyses of the lncRNA regulation. In this study, we built a knowledge base of human lncRNA functions in cancer, called lncRNAfunc. LncRNAfunc is the first database to systematically analyze the interaction between lncRNAs and different biomolecules across human pan-cancer and provides the potential lncRNA-related mechanisms and splicing regulation, which will be helpful to identify therapeutically targetable lncRNAs and potential functional roles in tumorigenesis.

For this aim, first, we analyzed the 15 900 lncRNAs expressed in The Cancer Genome Atlas (TCGA) across 33 cancer types and identified the tumor-specific lncRNAs/coding genes/exon skipping (ES) events by comparing gene expression and percent spliced in (PSI) values between tumors and matched normal samples. Next, to fully understand the key regulatory of lncRNAs, we performed multiple correlation analyses between lncRNAs and diverse biomolecules/cellular events including coding genes, alternative splicing events and miRNAs. Most of all, we intensively analyzed the interactions between lncRNAs and different types of biomolecules such as interactions with enhancers/promoters of the correlated genes in DNA-level, interactions with 3′-UTRs of the correlated genes, skipped exon regions of correlated alternative splicing events in RNA-level, interactions with the correlated miRNAs, and interactions with the potential regulators in protein-level such as transcription factors (TFs), splicing factors (SFs) and epigenetic factors (Epis). Furthermore, we also performed multiple functional annotations to identify cancer stage-associated lncRNA genes, potential miRNA binding sites in lncRNAs, RNA editing associated lncRNAs in cancer, expression quantitative locus (eQTLs) of lncRNAs, and single-nucleotide variant (SNV)-associated lncRNAs. Through our analyses, we identified 12 576 lncRNA-TF pairs, 7230 lncRNA–RNA binding protein (lncRNA-RBP) pairs, and 12 456 lncRNA-Epi pairs. These pairs showed co-expression patterns in more than five cancer types. Based on these integrative analyses, we identified 642 tumor-specific lncRNAs associated with 6433 differentially expressed coding genes, four tumor-specific lncRNAs associated with six exon skipping events. These lncRNAs need further validation of their roles in tumorigenesis. All these analysis results can be viewed and downloadable from the database with diverse biostatistical visualizations (https://ccsm.uth.edu/lncRNAfunc).

## MATERIALS AND METHODS

### Used datasets

To explore the regulating mechanism of lncRNAs, gene expression and alternative splicing, and sequence information are critical. We first downloaded the level 3 data of TCGA data including gene expression, miRNA expression and SNVs using TCGAbiolink (15) for 33 cancer types. Then, we collected alternative splicing event information of 32 cancer types of TCGA from Kahles et al.’s study (https://gdc.cancer.gov/about-data/publications/PanCanAtlas-Splicing-2018) (16). We download the lncRNA sequences, 3′-UTR region sequence of coding gene form GencodeV22 (17), and protein sequence from UniProt (18) to explore the interaction between lncRNA and multiple biomolecules.

### Identifying differentially expressed lncRNAs, coding genes, miRNAs and exon skipping events

To do our study, we downloaded expression values of ∼16K lncRNAs, ∼20K coding genes and ∼1900 miRNAs from the TCGA study. For each cancer type, we first filter out the lowly expressed genes based on the average value of the fragments per kilobase of exon per million mapped fragments (FPKM) <1 for lncRNAs and coding genes, and average reads per million (RPM) miRNA mapped <1 for miRNAs (Supplementary Table S1). After removing very low expressed genes, we used 6067 lncRNAs, 16 947 coding genes and 827 miRNAs in total. After investigation of the overlaps among cancers, we found 2645 lncRNAs that were expressed in only one cancer type, which might be related to cancer type-specific expressions. 3422 lncRNAs were expressed in more than two cancer types, which showed a common expression pattern in pan-cancer. Then to identify potential tumor-specific lncRNAs, coding genes and miRNAs, we ran DESeq2 (19) and perform differentially expressed lncRNAs/coding genes/miRNAs analyses between tumor and matched normal samples for individual cancer types, and identified differentially expressed lncRNAs (DElncRNAs), differentially expressed genes (DEGs) and differentially expressed miRNA (DEmiRNAs). Tumor-specific lncRNAs, coding genes and miRNAs were selected based on criteria of adjusted *P*-value <0.05 and |log_2_FC| >1. To explore cancer-stage associated lncRNAs, we also performed an association study between lncRNA expression and cancer stage information (|*R*| > 0.3 and adjusted *P*-value < 0.05). For differential exon skipping events (DEexs) between tumor and matched normal samples, Wilcoxon test and following Benjamini–Hochberg false discovery rate for multiple testing were applied to the percent spliced in values (|ΔPSI| > 10% and adjusted *P*-value < 0.05) to identify tumor-specific exon skipping events. Last, to infer the potentially involved biological processes of lncRNAs and coding genes, we used lncSEA (20) and Enrichr (21) for DElncRNAs and DEGs.

### Identifying lncRNA-associated coding genes/miRNAs/exon skipping events

For the relationship studies between lncRNAs and coding genes, miRNAs, we performed Pearson correlation association studies between lncRNAs’ gene expression and coding genes/miRNAs expression. Then, the Benjamini-Hochberg false discovery rate was used for multiple testing (|*R*| > 0.3 and adjusted *P*-value < 0.05). For the correlation between lncRNAs and alternative splicing events, we performed the association studies between the expression of individual lncRNAs and PSI values of individual exon skipping events (|*R*| > 0.3 and adjusted *P*-value < 0.05). To explore the commonly shared regulations in pan-cancer, we investigated the lncRNA-gene pairs, lncRNA–miRNA pairs, and lncRNA-exon skipping event pairs that correlate in at least five cancer types.

### Identifying potential interactions between lncRNAs and proteins

To study the lncRNA–protein interactions, we downloaded the important regulator's protein sequences from UniProt (18), including 1622 transcription factors (22), 639 RNA binding proteins (23), and 820 epigenetic factors. To predict the binding affinity between lncRNAs and proteins using their sequences, we used lncPro (23) and catRAPID (24). Only the lncRNA–protein pairs indicated by both tools were left for the next step. From the correlation analyses, only the lncRNA-transcription factor pairs, lncRNA–RNA binding protein pairs, and lncRNA-epigenetic factors pairs that were positively correlated and interacted with each other were selected as the co-expressed lncRNA–protein complexes.

### Identifying potential interaction between lncRNAs and DNAs

The promoters and enhancers are the primary regulatory components of the genome, which are able to regulate gene expression by interacting with transcript factors and diverse regulatory molecules (25). We download the enhancer and promoter regions from the FANTOM (26), EnhancerAtlas (27), EPD (28). Then overlapped the enhancer regions to the upstream 2–5 kb and downstream 2–5 kb of transcription start site for individual genes. The overlapped regions were saved for individual genes and used for further analysis. Gene promoter regions were defined as the overlap regions between upstream and downstream 2 kb of transcription start site and the promoter regions in FANTOM/ EPD for each gene. To study the lncRNA-DNA interactions, we used Triplex Domain Finder (TDF) (29). For lncRNA–enhancer interactions, we used the software option of ‘regiontest’ to predict the binding affinity between lncRNA sequences and gene enhancer regions. For lncRNA–promoter interactions, we ran TDF with an option of ‘promotertest’ to predict the interaction between lncRNAs and gene promoters. Then lncRNA-enhancers/promoters pairs were selected if lncRNAs and genes interacted and correlated with each other.

### Identifying potential interaction between lncRNAs and RNAs

As one of the regulatory regions in the gene body, this time, we focused on 3′-UTRs due to the unique role of regulating mRNA stability. To study the lncRNA–mRNA interactions, we first downloaded the 3-′UTR sequences of coding genes from Gencode V22, then ran lncTar (30) to predict the binding affinity and target region between the lncRNA sequences and 3′-UTR sequences. We selected 3′-UTRs bound by lncRNAs with more than binding scores of 90. The lncRNA-associated 3′-UTR was identified as being targeted by lncRNAs and correlated expression with lncRNA expression. Furthermore, we also used lncTar (30) to identify the lncRNA-associated exon skipping events through identifying the skipped exon’s sequences that were targeted by lncRNAs and have a significant correlation between PSI values of exon skipping events and lncRNA expression.

### Identifying tripple regulatory among miRNAs, lncRNAs and mRNAs

Micro RNAs bind to lncRNAs and mRNAs to down-regulate the expression of RNAs. To uncover involved lncRNAs in this mechanism, we study the interaction between miRNA and lncRNA/mRNA. To do this, we ran miRanda (31) and detected the miRNA binding sites in reference lncRNA sequences and mRNA 3′-UTR sequences. Based on the prediction and association studies between miRNA expression and lncRNA/gene expression, miRNA-lncRNA/mRNA 3′-UTR pairs were identified as interacting and correlated per cancer type.

### Identifying experimental supported interactions

The lncRNAfunc checked the lncRNA interaction databases include ENCORI, the updated version of starBase (32), NPInter (12), RNAInter (33) and mirTarBase (34), which provided experimental validations from CLIP-seq (35), RIP-seq (36), etc. Since the category of each database is not very specific thus we merged all the interactions in these four databases, we overlapped our interaction results with the validated ones recorded in these other public resources using interacted gene-gene pairs as the comparison unit. Then we performed a hypergeometric test and calculated the false positive rate (FDR) for each category.

### Identifying effects of A-to-I RNA editing on the expression of lncRNAs

For all the samples across 33 cancer types, we detected RNA A-to-I editing events from individual RNA-seq bam files by running the script of REDItoolKnown.py (REDItools) (37). Then focused on the known RNA A-to-I editing sites from REDIportal (January 2021) (38), we removed possible Single nucleotide polymorphism data and filtered out the candidates with supporting reads under three or editing frequency less than 0.1. Then, we identified 412 779 A-to-I RNA editing events that happened in 3639 lncRNAs. Next, we analyzed differentially expressed genes between RNA A-to-I-edited tumor samples and non-edited tumor samples (|log2FC| > 0.8 and adjusted *P*-value < 0.05). We also analyzed the relationships between RNA A-to-I editing frequencies and host lncRNAs’ expression (|*R*| > 0.3 and adjusted p-value < 0.05) and identified 311 editing-associated lncRNAs.

### Expression quantitative locus (eQTLs) of lncRNAs and single-nucleotide variants associated lncRNAs

Single nucleotide polymorphisms (SNPs) and single-nucleotide variants (SNVs) also can affect the lncRNA expression. Therefore, to identify eQTLs, we used PancanQTL (39). The Cis-eQTLs of lncRNAs that SNPs within 1 Mb of the lncRNAs and *P*-value <10^–5^ were selected to study the effects of SNPs on lncRNAs expression. To study the effects of mutation in lncRNAs, We grouped the tumor samples as mutation versus nonmutation groups. Next, we analyzed differentially expressed genes between mutation tumor samples and nonmutation tumor samples (|log_2_FC| > 0.8 and adjusted *P*-value < 0.05).

### Coding potentials of lncRNAs

To analyze coding potentials of lncRNAs from their transcript sequences, we input the lncRNA transcript sequences (all lncRNA transcript sequences from Gencode v22) to open reading frame finder (ORFfinder) (40). We adopted the translated open reading frame (ORF) sequences and the position of start and end from ORFfinder outputs.

### Database architecture

The lncRNAfunc system is based on a three-tier architecture: client, server, and database. It includes a user-friendly web interface, a Perl's DBI module, and a MySQL database. This database was developed in MySQL 3.23 with the My-ISAM storage engine.

## WEB CONTENTS AND ANALYSIS RESULTS

### Overview of lncRNAfunc

**Figure 1.**
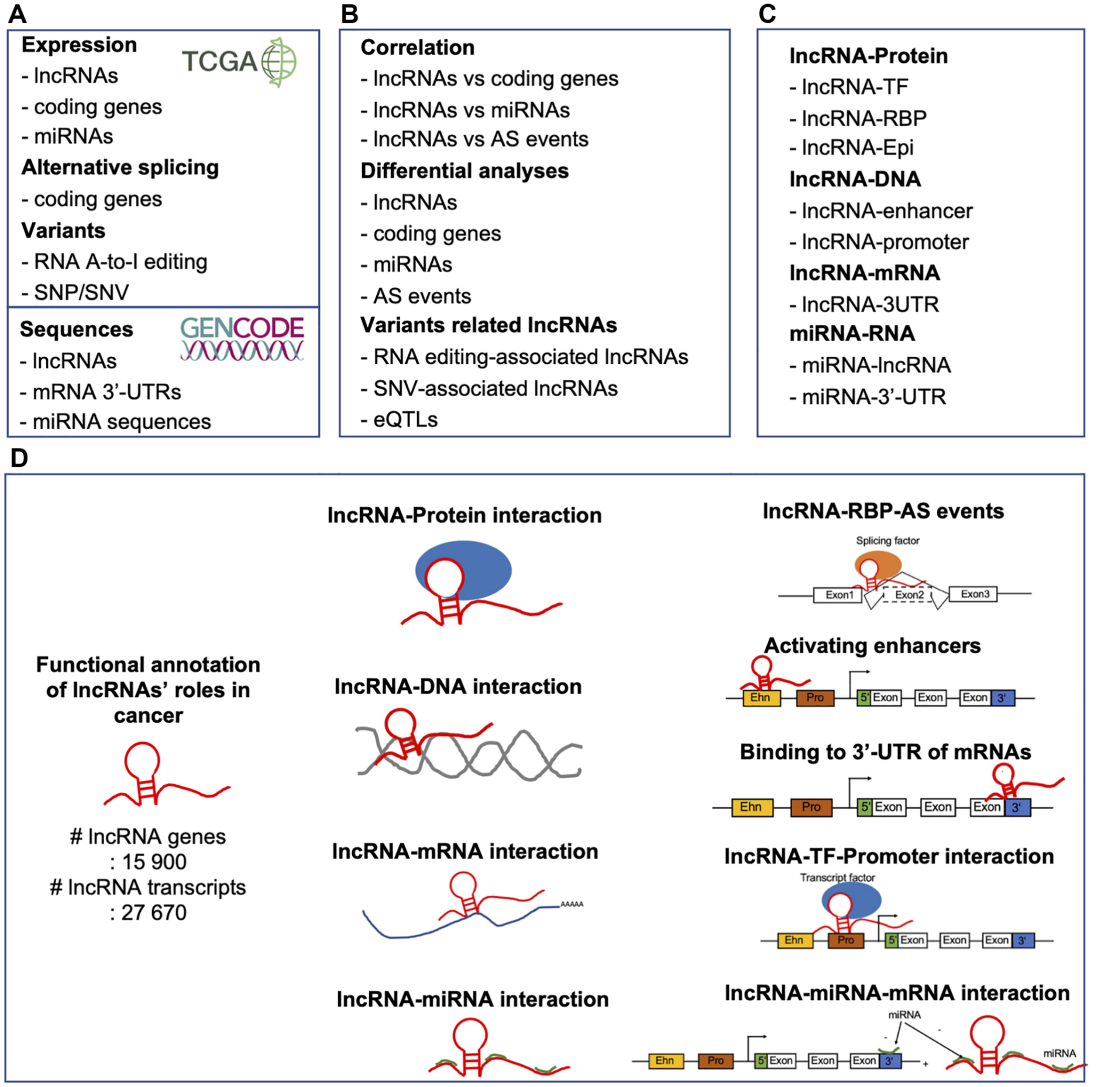
Overview of lncRNAfunc. (**A**) Data. (**B**) Basic analyses. (**C**) Interaction analyses. (**D**) Functional annotation of individual lncRNAs in lncRNAfunc.

lncRNAfunc provides ten categories of annotations on potential mechanisms of lncRNAs in human cancer (Figure 1). First, we compared the expression of genes between tumors and matched normal and identified 2525 potential tumor-specific lncRNAs. Among these tumor-specific lncRNAs, 1103 lncRNAs had correlations between their expressions and cancer stage information. Next, we performed correlation analyses and interaction analyses of lncRNAs with coding genes, alternative splicing events, and miRNAs. Our investigation of multiple types of interaction involving lncRNAs in human provides four potential mechanisms of expression regulation via lncRNAs as following: (i) 873 lncRNAs may regulate 6579 coding genes’ expression through targeting the 3′-UTR of mRNAs (78 057 lncRNA-3′-UTR pairs). (ii) 373 lncRNAs may regulate 5739 coding genes through functioning as enhancers (135 033 lncRNA-GeneEhn pairs). (iii) 213 lncRNAs may regulate the 3465 coding gene expression through recruiting 338 transcription factors (83 1339 lncRNA-TF-Gene triplets). (iv) 235 lncRNAs promote 2649 coding genes’ expression by competing for the binding sites of 103 miRNAs with these genes (21 388 lncRNA-miRNA-mRNA triplets).

One of the famous lncRNAs, MAGI2 antisense RNA 3 (*MAGI2-AS3*), inhibiting breast cancer cell growth by targeting the Fas/FasL signaling pathway in a previous study (41), were down-regulated in five cancer types in our study (Supplementary Figure S1A). Our pan-cancer interaction studies of *MAGI2-AS3* between diverse biomolecules found that *MAGI2-AS3* is associated with 2526 coding genes, which were enriched in cancer-pathways such as, ‘retinoblastoma gene in cancer’, ‘VEGFA-VEGFR2 signaling pathway’, and ‘pathways in clear cell renal cell carcinoma’ (Supplementary Figure S1B, C). Out of the *MAGI2-AS3* associated coding genes, 175 and 21 genes were positively correlated with *MAGI2-AS3* and showed differentially expressed between cancer and normal tissues which were expected to be promoted by *MAGI2-AS3* through being targeted their gene enhancers, recruiting TFs to the gene promoter. *MAGI2-AS3* also competing with the miRNA binding sites so that potentially protect mRNAs of nine DEGs. Moreover, *MAGI2-AS3* seemed negatively regulate with 103 through targeting 3′-UTRs of mRNAs. (Supplementary Figure S1D).

lncRNAfunc also provides the annotation on the potential regulatory mechanisms of lncRNAs with alternative splicing events. From our study of interactions and associations, we identified 113 lncRNAs regulating 46 exon skipping events through interacting with 70 RNA-binding proteins (745 lncRNA–RBP–ES triplets). We also performed more functional annotations and identified 311 RNA A-to-I editing-associated lncRNAs in the tumor, 5429 *cis*-eQTL pairs of 49 lncRNAs, 20 SNV-associated lncRNAs and lncRNA coding potential. These lncRNAs may have potential tumorigenic roles and may be worthy of further validation as the potential biomarkers and therapeutic targets in cancer. As explained here, lncRNAfunc provides intensive functional annotations of individual lncRNAs in human pan-cancer.

### lncRNAs regulate mRNA stability by binding to 3′-UTRs

**Figure 2.**
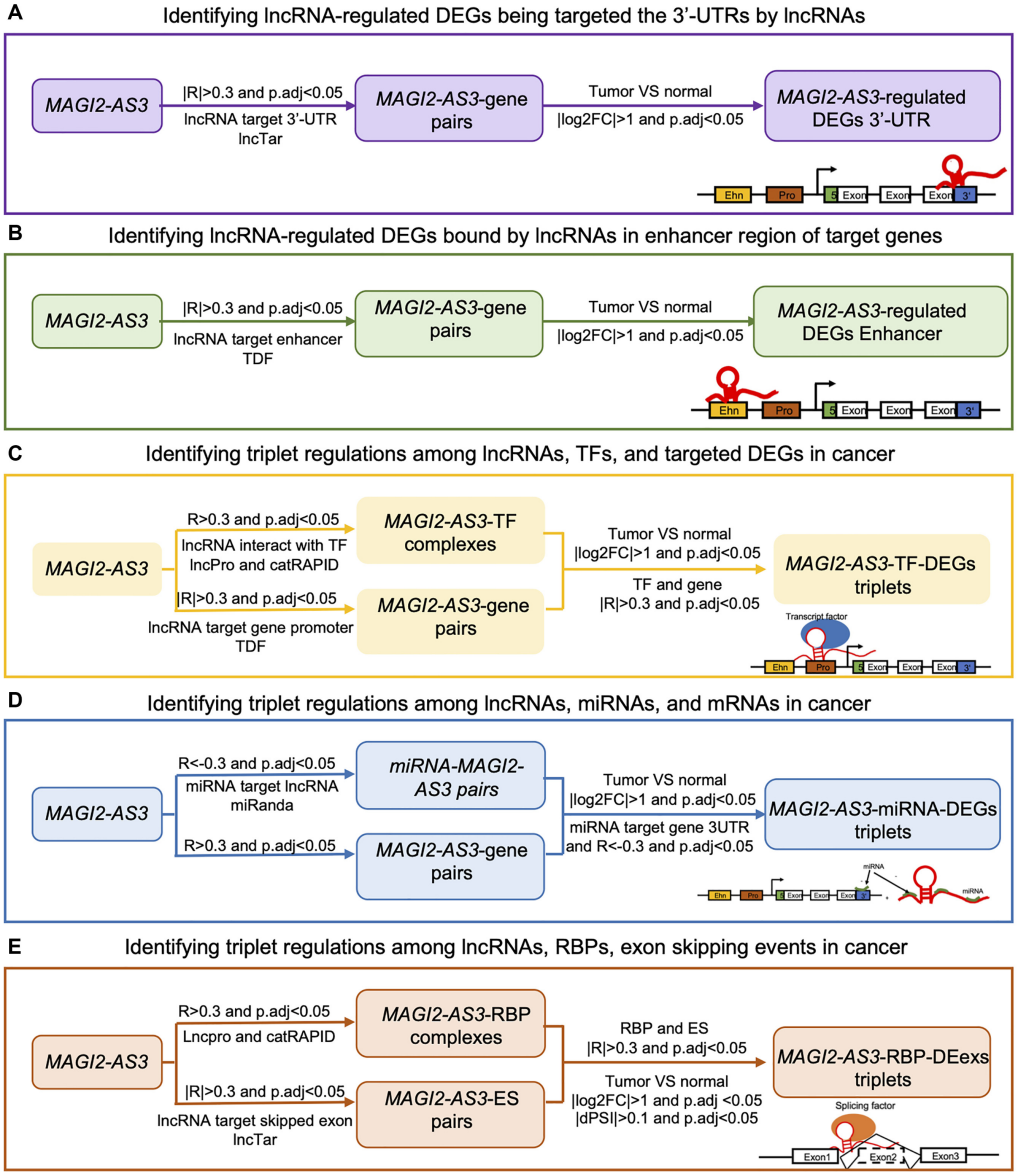
Pipelines to identify the cancer-related *MAGI2-AS3* regulation. (**A**) The pipeline to identify *MAGI2-AS3* associated DEGs through lncRNA–3′-UTR interaction. (**B**) The pipeline to identify *MAGI2-AS3* associated DEGs through lncRNA-gene enhancer interaction. (**C**) The pipeline to identify *MAGI2-AS3*-TF- DEG triplets. (**D**) The pipeline to identify *MAGI2-AS3–*miRNA–DEG triplets. (**E**) The pipeline to identify *MAGI2-AS3–*RBP–DEex triplets.

lncRNAs can interact with the 3′-UTR of mRNAs and decay the mRNA, which is one of the mechanisms that lncRNAs regulate gene expression (42). For example, half-staufen 1-binding site RNAs (*1/2-sbsRNAs*) bind to the Alu element of the 3′-UTR of target genes in the staufen 1 (*STAU1*)-mediated mRNA decay pathway, thus regulates mRNA degradation (43). To identify these lncRNAs regulating gene expression through binding to 3′-UTRs, we ran the lncRNA–RNA interaction prediction tool, LncTar (30). The association study between lncRNAs and coding genes, we found 6579 genes that were negatively correlated with 873 lncRNAs in more than 5 cancers, and 3′-UTRs were targeted by lncRNAs in 78 057 lncRNA-3′-UTR pairs. Furthermore, 8303 of 78 057 lncRNA-3′-UTR pairs showed differentially expressed patterns between tumors and matched normal samples. Out of these, 408 DELncRNAs and 2500 DEGs were involved in biological pathways of cancer progression (Supplementary Table S2). Then, we identified that *MAGI2-AS3* was associated with 103 DEGs and targeting the 3′-UTRs of these genes (Figure 2A). Functional enrichment analysis showed that these 103 genes were enriched in ‘VEGFA-VEGFR2 signaling pathway’, ‘pathways in clear cell renal cell carcinoma’, and ‘metabolic reprogramming in colon cancer’ by a gene set enrichment analysis web server, Enrichr (Supplementary Table S3). Previous studies showed that RecQ Like Helicase 4 (*RECQL4*) has oncogenic potential in sporadic breast cancers. Overexpression is associated with poor prognosis in patients with gastric cancer (44,45). In our study, *RECQL4* was also overexpressed in the stomach adenocarcinoma (STAD). Our association studies and interaction analyses found that *MAGI2-AS3* targets to 3′-UTR of *RECQL4* and has a negative correlation with *RECQL4*. This result may suggest that aberrant expression of *MAGI2-AS3* between tumors and normal samples might regulate the expression of *RECQL4* through targeting the 3′-UTR region and regulate the cancer progression (Supplementary Figure S1E and Supplementary Table S4). Overall, Our database provided all the lncRNA–3′-UTR interactions for pan-cancer which help users understand the regulation of lncRNAs for coding genes through targeting the 3′-UTR region.

### lncRNAs associated enhancers affect the genes’ expression in cancer

Previous studies found lncRNAs targeted the enhancer of genes and activate the gene expression, which was able to participate in important disease pathways and impact diverse human diseases (46,47). For example, leukemia-induced non-coding activating RNA 1(*LUNAR1*) can bind the intronic insulin like growth factor 1 receptor (*IGF1R*) enhancer element, recruiting the Mediator complex, and resulting in full activation of the *IGF1R* promoter (48). However, to date, there are no systematic analyses to identify the lncRNA regulated enhancers in cancer. For this aim, we ran TDF (29) to identify the lncRNA-gene enhancers interactions and performed associated study to identify the correlated lncRNA-Gene pairs in cancer. From our analysis, 32 2616 lncRNA–gene enhancer interaction pairs were discovered correlated with each other between 705 lncRNAs and 8222 coding genes. Among them, 37 915 lncRNA–gene enhancer pairs were related to the aberrant expression of coding genes between cancer and normal tissues. Since 346 DELncRNAs were predicted to bind to the enhancer region of 4859 DEGs, and DElncRNAs’ expression also showed a positive correlation with these DEGs. Functional enrichment analysis showed that these 870 coding genes were mainly involved in cancer-related pathways including ‘choline metabolism in cancer’, and ‘Rap1 signaling pathway’. Interestingly, *MAGI2-AS3* was positively correlated with 175 DEG’s expression and target to these gene enhancer regions (Figure 2B). One of them, Protein tyrosine phosphatase non-receptor type 13 (*PTPN13)* is an important regulator of tumor aggressiveness. It is known to regulate the aggressiveness of breast cancer cells through the inactivation of Src kinase from the previous studies (49–51). In this study, we found that *MAGI2-AS3* might interact with the enhancer of *PTPN13* and activate the expression of *PTPN13* in normal samples. This may support the down-regulated *MAGI2-AS3* may reduce the expression of *PTPN13* in cancer and give aggressiveness to cancer (Supplementary Figure S1F). Our analysis provided all the potential cancer-related lncRNA–GeneEnh pairs of 33 cancer types.

### lncRNA–TF–gene triplet relationship may provide novel regulation mechanisms in cancer

Transcription factors are the major regulators of target gene expression. lncRNAs interact with transcription factors in cancer could activate or repress gene expression (52,53). Such as long noncoding HIF-1α co-activating RNA (*LncHIFCAR*). A revious study found that *LncHIFCAR* acted as a hypoxia-inducible factor-1α(HIF-1α) co-activator driving oral cancer progression. *LncHIFCAR* formed a complex with HIF-1α via directly binding and facilitates the recruitment of HIF-1α and p300 cofactor to the target promoters (54). To understand the relationships between TFs and lncRNAs, we analyzed the interaction between these two biomolecules. Through the association studies, we identified 12 576 lncRNA–TF interaction pairs that were co-expressed in more than five cancer types (lncRNA–TF complexes). Next, we found 248 lncRNAs that have a significant correlation with TFs. From these individual studies, out of 83 1339 lncRNA–TF–GenePromoter triplets, 7915 were related to cancer progression and lncRNAs, TFs, and genes in triplets showed differential expression between cancer and normal in the specifical cancer types. 1088 differentially expressed coding genes seemed to be regulated by 509 lncRNA-TF complexes (99 DElncRNAs and 146 differential expressed TFs).

Our interaction study showed that *MAGI2-AS3* might interact with 106 TFs and form *MAGI2-AS3*-TF complexes and *MAGI2-AS3* were also predicted as targeting the promoters of 489 genes. Besides, 68 TFs were correlated with 252 coding genes. Finally, we identified 2119 *MAGI2-AS3–*TF–gene promoter triplets. Combining with DEG analysis result, a total of 21 DEGs were identified as being regulated by 12 *MAGI2-AS3–*TF complexes and were enriched in the biological processes such as ‘transcriptional misregulation in cancer’, ‘prostate cancer’, and ‘TGF-beta signaling pathway’ (Figure 2C, Supplementary Table S3). Like *MAGI2*-AS3–ZEB1– *ZEB1* triplets in BRCA (Supplementary Figure S2A). From our analyses, zinc finger E-box binding homeobox 1 (ZEB1), with UniProt (18) accession of P37275, was predicted to interact with two transcripts of *MAGI2-AS3* (ENST00000452320 and ENST00000617955). Association analysis between lncRNAs and TFs showed that *MAGI2*-AS3 and ZEB1 were positively correlated in nine cancer types (ACC, BRCA, KICH, KIRP, LUAD, PAAD, PRAD, STAD and UCS) (Supplementary Table S5). Endothelial PAS domain protein 1(*EPAS1*) was reported to promote peritoneal carcinomatosis of non-small-cell lung cancer by enhancing mesothelial–mesenchymal transition (55). Interaction analysis between *MAGI2-AS3* and the promoter region of *EPAS1* identified potential 178 interactions Top10 interactions were shown in Supplementary S4. What's more, *MAGI2-AS3, EPAS1* and ZEB1 show differential expression between cancer and normal samples in BRCA. All the evidence may suggest that *MAGI2-AS3* might promote the *EPAS1* expression through recruiting ZEB1 to the promoter of *EPAS1* then affects the cancer progression. Obviously, lncRNAfunc's lncRNA–TF–gene triplets will be helpful for users to identify the potential regulation by lncRNA and provide the therapeutic target.

### Analysis of lncRNA–miRNA–mRNA triplet relationship based on competitive endogenous RNA reveals functional lncRNAs in cancer

Competitive endogenous RNAs (ceRNAs) have revealed a new mechanism of interactions among diverse types of RNAs playing crucial roles in multiple biological processes and the development of neoplasms (56,57). *MALAT1* is one of the best examples of the lincRNAs as working as the ceRNAs. *MALAT1* has been reported as promoting signal transducer and activator of Transcription 3 (*STAT3*) expression by binding and sequestering its major negative regulator miR-125b in oral squamous cell carcinoma cells (58). lncRNAs can compete with mRNAs for the miRNA binding sites. To identify the lncRNA–miRNA–mRNA triplets, we first identified miRNA–lncRNA interaction pairs and miRNA–mRNA interaction pairs individually and then calculated the correlation between them. From the results, only the negatively correlated miRNA–lncRNA and miRNA–mRNA interaction pairs and positively correlated lncRNA–mRNA interaction pairs were used to build these triplets. Finally a total of 21 388 lncRNA–miRNA–mRNA triplets were identified and 3752 lncRNA–miRNA–mRNA triplets of them were found as being related to cancer progression by comparing the expressions of lncRNAs, miRNAs and mRNAs between cancer and normal samples. There were 128 DElncRNAs, 76 DEmiRNAs, and 1045 DEGs. Taking *MAGI2-AS3* as an example, 10 lncRNA-miRNA-mRNA triplets used *MAGI2-AS3* as the ceRNAs with 2 miRNAs (hsa-mir-96 and hsa-mir-182) and nine genes (Figure 2D). Interestingly, hsa-mir-96 was negatively correlated with these nine genes in BRCA and functionally enriched in the ‘TGF-beta signaling pathway’, ‘pathways in clear cell renal cell carcinoma’, and ‘integrated breast cancer pathway’ (Supplementary Table S3). Decorin (*DCN*) participated in ‘TGF-beta signaling pathway’ and it was down-regulated in BRCA. A previous study reported that *DCN* suppressed tumorigenesis, invasion, and metastasis in inflammatory breast cancer (59). In our study, the hsa-mir-96 was predicted to bind to both of 3′-UTRs of *MAGI2-AS3* and *DCN*, which indicates *MAGI2-AS3* might protect *DCN* through competing for the miRNA binding sites of hsa-mir-96 (Supplementary Figure S2B, Supplementary Table S6). In this study, we identified potential ceRNA regulation, which might provide insights for further research on the molecular mechanism and potential prognosis biomarkers.

### lncRNAs affect the exon skipping events by recruiting RNA binding proteins (splicing factors)

lncRNAs are involved in the mechanism of alternative splicing events in several ways. lncRNAs can regulate the gene splicing process through interacting with splicing factors, which are the major RNA binding proteins (60). For example, *MALAT1* is able to regulates alternative splicing by modulating arginine-rich (SR) splicing factor phosphorylation. Depletion of *MALAT1* might changes the alternative splicing of a subset of transcripts (61). Through investigating the interactions between lncRNAs and RNA binding proteins (RBPs), we identified 7230 potential lncRNA–RBP complexes, which had interactions between lncRNAs and RBPs and positively correlated between them in more than five cancer types. Exon skipping (ES) is the most common type of multiple types of alternative splicing events with the effects of potential loss of functional domains/sites or frameshifting of the ORF. Based on this context, we performed the associated study to identify the lncRNA-associated ES events through calculating the correlations between lncRNAs’ expression and exon skipping events’ PSI values. We also identified lncRNA targeted ES events through predicting the interactions between skipped exon regions of individual ES events and lncRNAs. Only the interacted and correlated lncRNA-ES pairs were selected to construct the lncRNA–RBP–ES (exon skipping) triplets. Through these analyses, finally, 745 lncRN–RBP–EX triplets were identified. Out of these, seven triplets showed significant association with the cancer progressions. These findings include four DElncRNAs, three differentially expressed RBPs and six DEexs of six genes.

Another example is *MAGI2-AS3*, which interacted with RNA binding motif single stranded interacting protein 3 (RBMS3) and formed *MAGI2-AS3-*RBMS3 complex (Figure 2E, Supplementary Figure S3C). In PRAD, we found the interactions between this complex and the skipped exon regions of four genes. These four exon skipping events include RNA binding motif protein 26 (*RBM26*), myosin XVIIIA (*MYO18A*), LRR binding FLII interacting protein 2 (*LRRFIP2*), and NUMB endocytic adaptor protein (*NUMB*) (Supplementary Table S6). Among them, *RBM26* (ExonSkipDB’s exon skipping ID: exon_skip_104174), *MYO18A* (exon_skip_287874) and *LRRFIP2* (exon_skip_382356) were positively correlated with *MAGI2-AS3* and skipped these specific exons in PRAD. However, *NUMB* (exon_skip_114200) was negatively associated with MAGI2-AS3 (Supplementary Figure S3A and B). Based on the functional annotation from ExonSkipDB (62), our previous work, these four exon skipping events were anticipated to form the in-frame ORFs (Supplementary Figure S3F). The exon_skip_287874 was predicted to cause the loss of the ‘Coiled coil’ protein domain of *MYO18A*. The exon_skip_382356 might cause loss of function of the ‘Coiled coil’ and ‘DVL3-binding’ protein domains of *LRRFIP2* (Supplementary Table S6). These exon skipping events in cancer patients may affect the normal cell progression and aggressiveness of cancer. *MAGI2-AS3*′s aberrant expression in cancer patients through the lncRNA–RBP–ES triplets may be related to cancer progression. As shown in these examples, lncRNAfunc will help users to better understand how lncRNA regulates alternative splicing in cancer.

### Public resources supported lncRNA interactions in lncRNAfunc

The experimental validations are essential to support our predicted interactions between lncRNAs and other molecules. However, there were no available RNA molecules’ interaction related sequencing experiments studies in TCGA. Therefore, we checked the several lncRNA interaction databases that include experimental validations from CLIP-seq, RIP-seq (36), ChIRP-seq (63), etc. Thus, we merged the interactions in ENCORI (The updated version of starBase) (32), NPInter (12), RNAInter (33), mirTarBase (34) (Supplementary Table S7). We overlapped our interaction results with the validated ones recorded in these public resources. From these analyses, we could identify 135 lncRNA-mRNA interactions, 25 lncRNA-miRNA interactions, 506 mRNA–miRNA interactions, 22 lncRNA–gene promoter interactions, 43 lncRNA–gene enhancer, and 3549 lncRNA–protein interactions in both lncRNAfunc and at least one of upper mentioned public resources. We divided the interactions into three categories including experimental validation, computational prediction, and literature mining-based evidence from these four resources. 135 lncRNA–mRNA interactions, 21 lncRNA–miRNA interactions, 48 lncRNA–DNA interactions and 2597 lncRNA–protein interactions were supported by experimental validation (Supplementary Figure S4). From our analysis, only lncRNA–DNA interaction seemed not significant. This might be due to the limited focused regions of genes like promoter or enhancer regions in our study, but other databases might include interactions in the whole regions of genes.

lncRNA HOXA Transcript Antisense RNA, Myeloid-Specific 1 (*HOTAIRM1*) has been reported as interacting with Homeobox A1 (*HOXA1*) in DNA level from RNAInter and lncRNAfunc. From the previous study, *HOTAIRM1* was reported as being able to affect the histone modification and methylation in the *HOXA1* promoter region then regulate the expression of *HOXA1* (53,64). In our study, *HOTAIRM1* was predicted as interacting with enhancer region and positively correlated with HOXA1 in ten cancer types. Both *HOTAIRM1* and *HOXA1* were also differentially expressed in ESCA and KIRP (Supplementary table S8). As shown in these findings, our prediction of interactions between RNA and other molecules with following correlation analyses is useful to identify the potential regulations of lncRNAs.

### RNA A-to-I editing events in lncRNAs

RNA A-to-I editings are frequent editing events in humans that can make a single base change on a specific nucleotide sequence in an RNA transcript. In this paper, we performed association studies between RNA editing frequency and their host lncRNA’s expression. Then, we identified 1654 RNA A-to-I editing events that were associated with 311 lncRNAs. To systematically analyze the potential contributions of these RNA A-to-I editing events to the expression levels of lncRNA genes, we performed the differential expression analysis between RNA-edited and non-edited tumor samples. There were 4037 and 100 editing events that would cause the up-/down-regulation of 376 and 35 lncRNAs, respectively. This result was combined with the DELncRNAs between cancer and the matched normal. We identified 895 and 5 editing events that would upregulate or downregulate the gene expression, respectively. These editing events that cause or increase the aberrant expressions in cancer, they may have the potential to worsen the cancer condition. Furthermore, there were 129 and 10 RNA A-to-I editing events up- or down-regulate the gene expression of 23 and 7 lncRNAs in cancer, respectively. (Supplementary Figure S5A). Indeed, 131 RNA A-to-I editing event-associated DElncRNAs were enriched in diverse biological processes of cancer like ‘ovarian cancer’, ‘colorectal carcinoma’, ‘renal clear cell carcinoma’, ‘colorectal carcinoma’, ‘osteosarcoma’, ‘renal clear cell carcinoma’, ‘multiple myeloma’, ‘glioma’, ‘non-small cell lung cancer’, ‘lung_cancer’ (Supplementary Figure S5B). One interesting example is HNF1A Antisense RNA 1 (*HNF1A-AS1*), which has been reported as promoting the cell-cycle progression in gastric cancer (65). There was a report about the deletion of this gene suppressed the malignant phenotypes of breast cancer cells (66). From our analysis, *HNF1A-AS1* was upregulated in STAD and 2476 RNA A-to-I editing events were detected in *HNF1A-AS1*. 55 of these events were correlated with *HNF1A-AS1* and affect *HNF1A-AS1* upregulated in edited STAD samples compare to non-edited STAD samples. These editing events seem to increase the gene expression abnormally in cancer, which may have the potential to aggravate the cancer progression (Supplementary Table S9).

It is noteworthy that RNA editing sites in chr7: 104987421, 105007031 and 105007757 (lncRNAfunc IDs: Lncediting_190011, Lncediting_190543, and Lncediting_190615) showing the potential influence on the expression of *LINC01004* in STAD (Supplementary Figure S5C, D). *LINC01004* might affect 70 DEGs through two lncRNA-3′-UTR interactions and 68 lncRNA-gene enhancer interactions, respectively. Functional enrichment analysis showed that these genes were involved in the cancer-related pathways, such as ‘ncRNAs involved in Wnt signaling in hepatocellular carcinoma’, ‘retinoblastoma gene in cancer’ and ‘lncRNA involvement in canonical Wnt signaling and colorectal cancer’ (Supplementary Table S10). These evidence may support the fact that RNA A-to-I editing events may work as upstream variants that affect the lncRNAs’ expression and involve cancer-related pathways. All information on the RNA editing events which might cause the aberrant expression of lncRNAs is available on the lncRNAfunc website.

### The effect of single nucleotide polymorphisms and single-nucleotide variant in lncRNAs

In addition to the RNA A-to-I editing event, the single nucleotide polymorphisms in lncRNAs or located near to lncRNAs and the SNVs in lncRNAs also can affect the expression of lncRNAs. For the SNPs associated with lncRNAs, we have downloaded the eQTL analysis results from PancanQTL (39) and identified 5429 *cis*-eQTL pairs with 49 lncRNAs, and 168, 224, 552 SNPs in the promoter, enhancer, and gene body of 28, 27, 24 lncRNAs, respectively. The differential analysis identified 11 DELncRNAs that were associated with 999 SNPs. These DELncRNAs were functionally enriched in ‘gastric cancer’, ‘chronic myelogenous leukemia’, ‘gastric cancer’, ‘bladder cancer’, ‘prostate cancer’, ‘lung adenocarcinoma’, and ‘esophageal cancer’ (Supplementary Figure S5E). 44, 30 and 40 c*is*-eQTL pairs happened in the promoter, enhancer and gene body of DELncRNAs, respectively (Supplementary Figure S5F). For example, lncRNA small nucleolar RNA host gene 7 (*SNHG7*) can promote the proliferation and inhibit apoptosis of gastric cancer cells (67). The eQTL analysis found four SNPs (rs6874, rs115850137, rs61749087 and rs72761015) in the enhancer region of *SNHG7* and two SNPs (rs72761016 and rs11145848) in the promoter region of *SNHG7*. These were associated with the *SNHG7* expression in five cancer types (Supplementary Table S11). To explore the effects of SNVs in lncRNAs, we performed DELncRNAs between mutated group and non-mutated group, and identified 20 SNV-associated lncRNAs including 7 lncRNAs and 13 mutations. One example is LncRNA CD27 antisense RNA 1(*CD27-AS1*), which is related to acute myeloid leukemia progression (68). In our study, we found that one mutation (chr11:65269611: -: TTTCC, Supplementary Table S12) in *CD27-AS1* might up-regulate the expression of mutation tumor samples in SARC. Overall, LncRNAfunc provides the upstream variants which might affect the lncRNAs’ expression which might affect downstream regulation of lncRNAs and cancer progression. All filtered *cis*-eQTL for lncRNAs and SNV-associate lncRNAs were shown in our database.

## DISCUSSION AND FUTURE DIRECTION

The user-friendly web interface of lncRNAfunc provides comprehensive analyses results on potential mechanisms of transcriptional regulation of individual lncRNAs in human cancer. These study results will be helpful to genetics, cell/molecular biology, and bioinformatics research communities. We admit that lncRNAs are not only involved in transcriptional regulation but also chromatin remodeling and post-transcriptional regulation. However, in this study, we mainly focused on the effects of lncRNAs on the transcription level by analyzing the interaction and expression with the coding genes, miRNAs, and exon skipping events due to the lack of chromatin structure data (i.e. Chip-seq (69), ATAC-seq (70), DNase-seq (71)) in TCGA. From the DEG analyses, we identified 2525 tumor-specific lncRNAs. The aberrant expression of these lncRNAs might affect the coding genes’ expression and alternative splicing through multiple interactions with different biomolecules, which might disturb the cancer progression. Based on our comprehensive analyses of interactions and correlations, we identified four potential regulations that might result in cancer aberrant coding gene expression via lncRNAs such as 8303 lncRNA-3′-UTRs, 37 915 lncRNA-gene enhancers, 7915 lncRNA-TF-Gene and 3752 lncRNA–miRNA–mRNA regulations. Seven lncRNA–RBP–ES triplets provide evidence of how lncRNAs can cause loss of function of the coding genes (Table 1). Since there were no available RNA molecules’ interactions related sequencing experiments TCGA study. We search the public experimental supported interactions resources to improve the reliability of our database. Totally 135 lncRNA–mRNA interactions, 25 lncRNA–miRNA interactions, 506 mRNA–miRNA interactions, 22 lncRNA–gene promoter interactions, 43 lncRNA-gene enhancer and 3549 lncRNA-protein interactions in lncRNAfunc had been reported in at least one resource. Upstream regulators were also considered in our studies (i.e. RNA editing and SNPs), which affect the lncRNAs expression including 1027 RNA editing events associated with 131 DElncRNAs and 999 *cis*-eQTL pairs in 11 DElncRNAs. LncRNAfunc aimed to provide diverse views of potential mechanisms involving lncRNAs in pan-cancer with potential interactions. In the future, we will explore the epigenetic regulations of lncRNAs by searching more available data of cancer and improve our approach to find clinically significant lncRNAs and downstream target genes. To improve the reliability of the predicted lncRNA regulations in cancer. We will keep searching the experimentally validated interactions of lncRNAs and update them to the database. Besides, public CLIP-seq/RIP-seq/ChIRP-Seq data sets might need to analyze to increase the reliability of the predicted lncRNA regulations in cancer. We hope we can systematically and intensively analyze big-size data of these high throughput data and find critical evidence from there in the future. To make better use of lncRNAfunc, we will add the analyses results for the lncRNAs of over 1000 cell lines from CCLE, as well as data sets for multiple mouse tissues. We believe lncRNAfunc will be a useful resource for diverse study fields of pathology, cancer genomics, and precision medicine research communities.

**Table 1.** Summary of cancer-related lncRNA regulation

	Positive regulation				Negative regulation			
lncRNA–TF–gene	#Triplets	#DELncRNA	#DETF	#DEG	#Triplets	#DELncRNAs	# DETF	#DEG
	7752	96	145	1058	161	10	28	41
lncRNA–RBP–ES	# Triplets	#DELncRNA	#DERBP	#DEex (Gene)	#Triplets	#DELncRNAs	#DERBP	# DEex (Gene)
	3	1	1	3(3)	4	4	3	3(3)
lncRNA–miRNA–mRNA	#Triplet	#DELncRNA	#DEmiRNA	#DEG				
	3752	128	76	1045				
lncRNA–GeneEnh	#Pairs	#DELncRNA		#DEG				
	37 915	346		4859				
lncRNA–3′-UTR					#Pairs	#DELncRNAs		# DEG
					8303	408		2500

* DE represent differential expression between tumor and normal tissues.

## Supplementary Material

gkab1035_Supplemental_Files
