# Supplementary material for: lncRNAfunc: a knowledgebase of lncRNA function in human cancer
Source: Nucleic Acids Res. 2021 Nov 17;50(D1):D1295–306. doi: 10.1093/nar/gkab1035 (PMC8728133; doi:10.1093/nar/gkab1035)
Supplement: gkab1035_Supplemental_Files [file gkab1035_supplemental_files.zip › lncRNAfunc_supplementary.pdf]

lncRNAfunc: a knowledgebase of lncRNA function in human cancer

Mengyuan Yang[1,2,3,†], Huifen lu[1,2,†], Jiajia Liu[3], Sijia Wu[4], Pora Kim[3,*], and Xiaobo Zhou[3,5,6,*]

[1] West China Biomedical Big Data Center, West China Hospital, Sichuan University，Chengdu 610041, China

[2] Med-X Center for Informatics, Sichuan University, Chengdu 610041, China

[3] Center for Computational Systems Medicine, School of Biomedical Informatics, The University of Texas Health Science Center at Houston, Houston, Texas, 77030, USA

[4] School of Life Sciences and Technology, Xidian University, Xi'an, 710126, China,

[5] McGovern Medical School, The University of Texas Health Science Center at Houston, Houston, TX 77030, USA

[6] School of Dentistry, The University of Texas Health Science Center at Houston, Houston, TX 77030, USA

*Address correspondence to:
Xiaobo Zhou, Ph.D. and Pora Kim, Ph.D.
School of Biomedical Informatics
The University of Texas Health Science Center at Houston
7000 Fannin St., Houston, TX 77030
Phone: 713-500-3923 and 3636
Email: Xiaobo.Zhou@uth.tmc.edu and Pora.Kim@uth.tmc.edu

[†]These authors contributed equally to this work.

Supplementary Figures

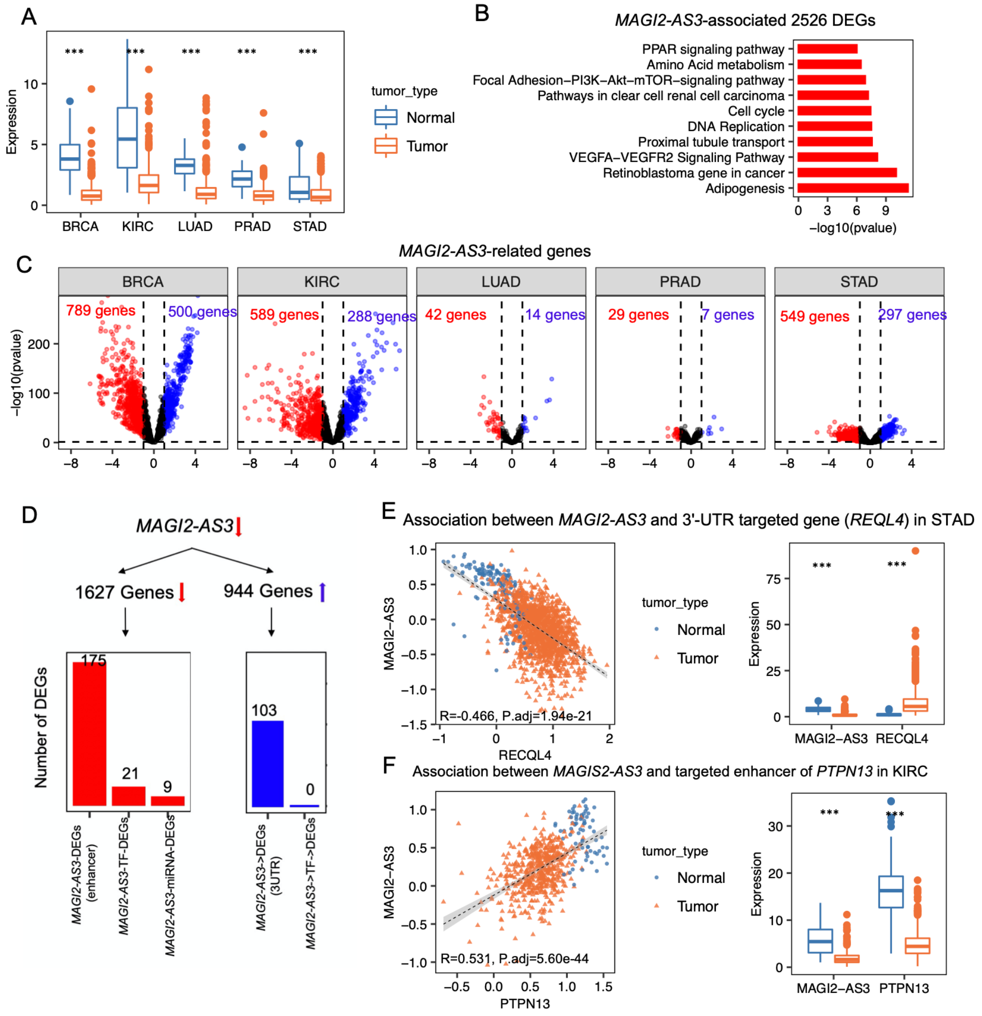

Supplementary Figure 1. *MAGI2-AS3*-associated DEGs regulation. (A) The boxplot of *MAGI2-AS3* in five cancers. (B) The enrichment pathway for *MAGI2-AS3*-associated 2526 DEGs. (C) The Volcano for all *MAGI2-AS3*- associated DEGs in five cancer types. (D) The summary figure for *MAGI2-AS3*-associated DEGs expression regulation. (E) *MAGI2-AS3* target 3'-UTR of RECQL4 in STAD. Left: gene expression correlation. Right: the boxplot of *MAGI2-AS3* and *RECQL4* expression in STAD. (F) *MAGI2-AS3* is an active the enhancer of *PTPN13* in KIRC. Left: gene expression correlation. Right: the boxplot of *MAGI2-AS3* and *PTPN13* expression in KIRC.

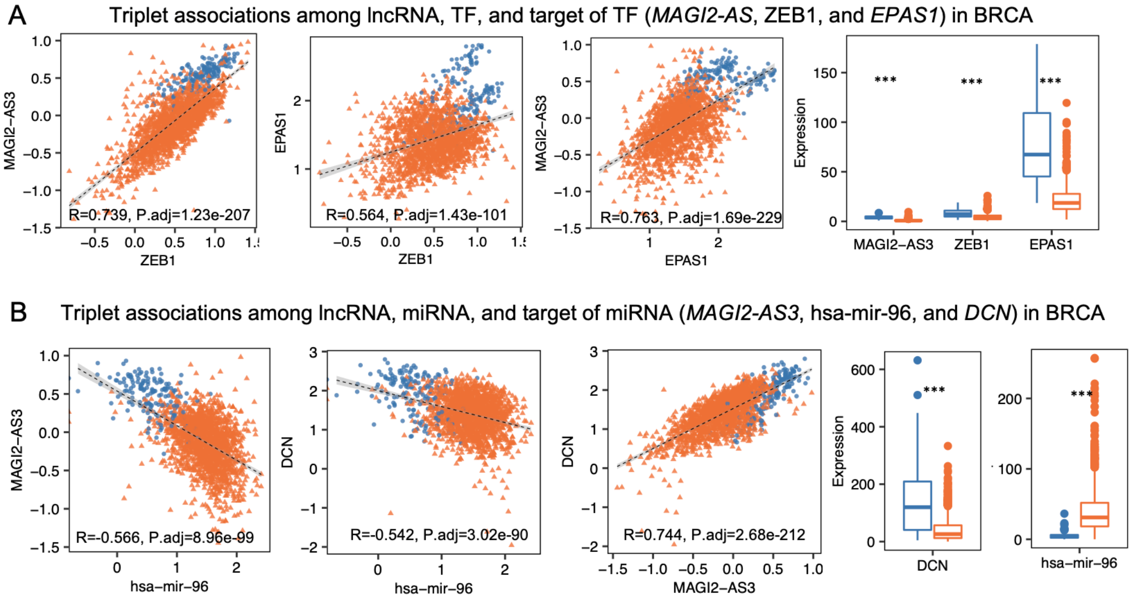

Supplementary Figure 2. (A) *MAGI2-AS3*-ZEB1-*EPAS1* triplet in BRCA. The left three figures are for the gene expression correlation. Last one is the boxplot of *MAGI2-AS3, ZEB1,* and *EPAS1* expression in BRCA. (B) The *MAGI2-AS3*-hsa-mir-96-*DCN* triplet in BRCA. The left three figures are for the gene expression correlation. Left fourth and fifth figures are the boxplots of expression of *DCN* and hsa-mir-96 in BRCA.

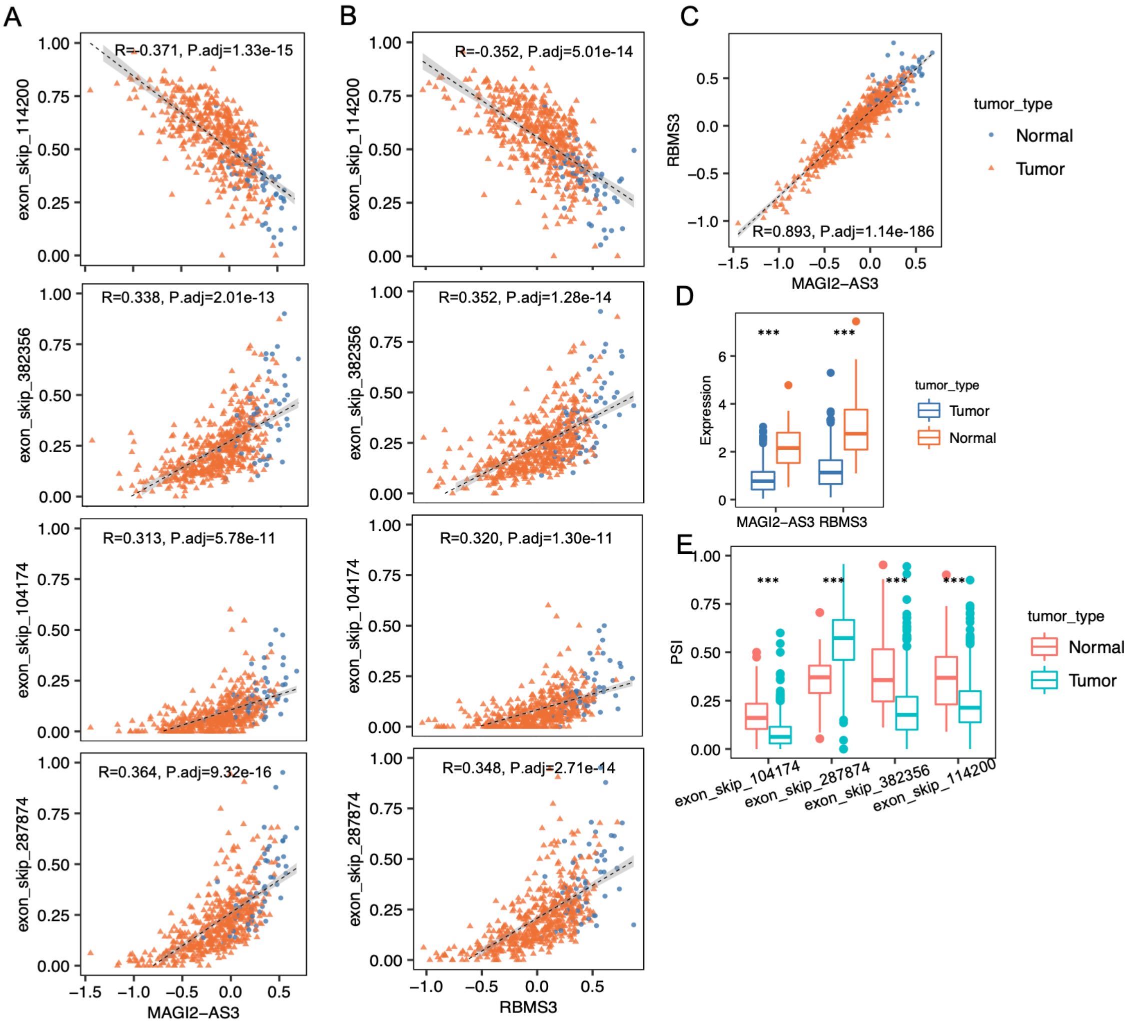

F ORF annotation of individual exon skipping events that are associated with *MAGI2-AS3* expression

Exon Skipping id	Skipped Exon	EX Gene Name	EX Affected Trans	ORF_anno
exon_skip_104174	chr13:79353152-79353224	RBM26	ENST00000438737	In-frame
exon_skip_287874	chr17:29085603-29085648	MYO18A	ENST00000527372	In-frame
exon_skip_382356	chr3:37091466-37091538	LRRFIP2	ENST00000336686	In-frame
exon_skip_114200	Chr14:73279280-73279424	NUMB	ENST00000355058, ENST00000555238	In-frame

Supplementary Figure 3. *MAGI2-AS3* related alternative splicing regulation in cancer. (A) Correlation between *MAGI2-AS3* expression and four associated exon skipping events PSI values in PRAD. (B) Correlation between *RBMS3* expression and four associated exon skipping events PSI values in PRAD. (C) Correlation between *MAGI2-AS3* expression and *RBMS3* expression in PRAD. (D) The boxplot of *MAGI2-AS3* and *RBMS3* expression in BRCA. (E) The boxplot of three *MAGI2-AS3* positive correlated exon skipping events' PSI values in BRCA. (F) ORF annotation of individual exon skipping events that are associated with *MAGI2-AS3* expression.

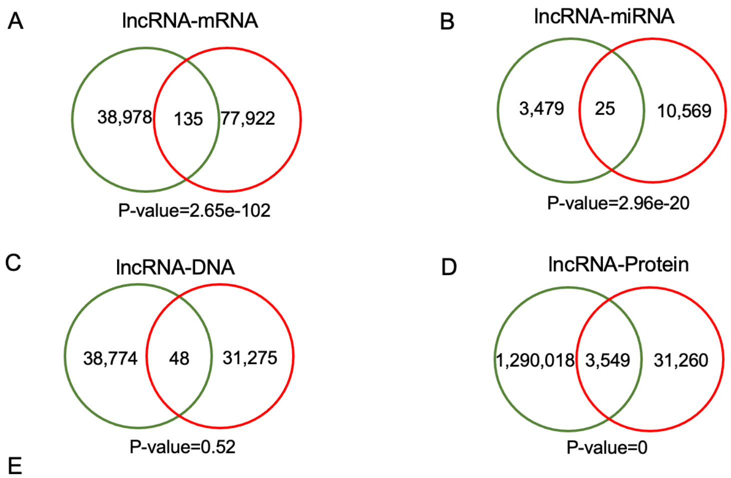

E

	#Interactions in lncRNAfunc	#Interactions supported by public resource				Hypergeometric test p-value
		Experimental validation	Literature mining	Computational prediction	Sum	
lncRNA-mRNA	39 113	135	-	-	135	2.65E-102
lncRNA-miRNA	3 504	21	1	2	25	2.96E-20
lncRNA-DNA	38 792	48	-	-	48	0.52
lncRNA-protein	1 293 567	2597	7	967	3549	0.00

Supplementary Figure 4. Comparison of multiple interactions between lncRNAfunc and four lncRNA interaction resources. The green and red circles represent the number of interactions in other databases and lncRNAfunc, respectively. (A) Interactions between lncRNAs and mRNAs. (B) Interactions between lncRNAs and miRNAs. (C) Interactions between lncRNAs and DNAs. (D) Interactions between lncRNAs and proteins. (E) The number of nteractions overlapped differential sources.

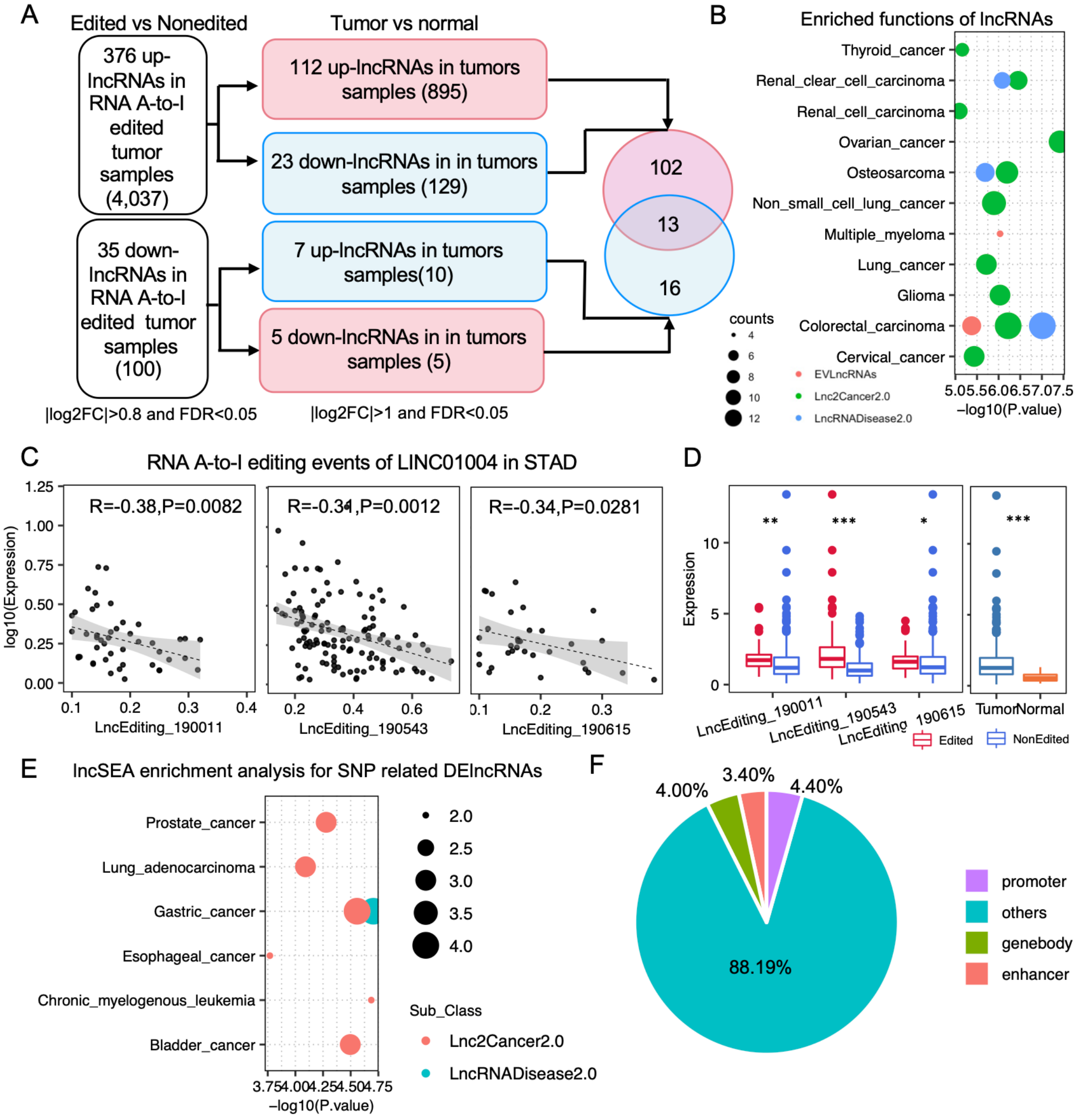

Supplementary Figure 5. The effects of RNA editing events on lncRNA expression. (A) Selection of cancer-related DELncRNAs (B) lncSEA enrichment analysis. (C) RNA editing of *LINC01004* in STAD. Correlations between editing levels and *LINC01004* expressions. (D) Differences of *LINC01004* expressions between RNA-edited and non-edited groups, and between AD and control groups.(E) lncSEA enrichment analysis for SNP related DELncRNAs. (F) The pie chart for a cis-SNP position.

Supplementary Tables

Supplementary Table S1. Number of lncRNA, protein coding gene and miRNA for each cancer type.

Supplementary Table S2. Cancer-related lncRNA-3'-UTR pairs.

Supplementary Table S3. Functional enrichment of *MAGI2-AS3*-associate DEGs.

Supplementary Table S4. *MAGI2-AS3* targeted *RECQL4* 3'-UTR regions.

Supplementary Table S5. Evidences of *MAGI2-AS3*-ZEB1 complex regulate *EPAS1* expression.

Supplementary Table S6. Cancer related *MAGI2-AS3*-RBMS3-ES triplets.

Supplementary Table S7. Interaction information from public resource.

Supplementary Table S8. The correaltion of *HOTAIRM1* and *HOXA1*.

Supplementary Table S9. RNA editing events related to *HNF1A-AS1*'s expression.

Supplementary Table S10. *LINC01004* associate DEGs.

Supplementary Table S11. Cis-eQTLs of *SNHG7*.

Supplementary Table S12. SNV associated lncRNAs.
